# Treatment of Typhoid with Remedies Other Than Drugs

**Published:** 1911-09

**Authors:** M. McH. Hull

**Affiliations:** Atlanta, Ga.


### TREATMENT OF TYPHOID WITH REMEDIES OTHER THAN DRUGS.

M. McH. Hull, M. D., Atlanta, Ga.

The information which has been gained from a clinical and laboratory study of a given case having established the diagnosis of typhoid fever, the following problem is presented to the therapeutist: He has to deal with an acute disease of long duration, due to the bacillus typhosus, with lesions in the lower ileum. A toxemia, and also a bacteremia is present, which produces great waste of tissue, elevation of temperature, weakness of all the muscles, especially the heart muscle, and certain definite symptoms associated with the nervous system.

It is our function to confine ourselves to those remedies other than drugs which are at the disposal of the physician to cambat this condition. It is scarcely necessary to say, and yet it is necessary to say, that it is the patient, not the disease, that we have to treat; hence the various remedies suggested will have to be modified to suit the individual case.

What remedies other than drugs are at our disposal to help us solve this problem? Naming them in the order of their relative importance, we have rest, diet, hydrotherapy, serum therapy, mechanotherapy. Let us give some attention to each of these.

For lack of time, four of these must be discussed in very few words here.

Rest in bed is an absolute necessity, to conserve the patient's strength for future needs, to prevent auto-inoculation, and to avoid fatigue which would result in excessive irritability. The patient should do nothing for himself that someone else can do for him, except the case be very mild.

Mechanotherapy is indicated as gentle friction given in connection with the baths as hereinafter mentioned.

The effect of light as a remedial measure is not usually given the importance it deserves. Light stimulates cellular activity and increases resistance, a moderate amount of light is helpful rather than harmful. Of course the patient should not be in the direct glare of the sun; neither should he be in a darkened room as we so often find.

Psychism holds its place in the treatment of this affection by reason of the stimulus which cheer and hope and confidence produce. These are developed in the patient by the proper attitude of the physician. While no false hopes should be held out to the patient, a frank statement of his condition, laying emphasis on the encouraging things, will do much to help turn the tide in favor of recovery. "A merry heart doeth good like a medicine" in typhoid as well as in other troubles.

The other three remedial measures mentioned, diet, hydrotherapy and serotherapy are far more important; these three, and the greatest of these is diet. In selecting a diet for a patient with typhoid fever, there are certain general principles which must be followed: 1st. We must remember that in every febrile condition, regardless of the cause, there is disordered digestion, due to diminished secretion of gastric juice and decreased motility of the stomach.. The powers of absorption are also diminished. If the febrile condition is of very short duration this element is not very important, but in a disease like typhoid where the duration is so long, it plays a very important part. 2nd. The presence of ulcers in the lower ileum makes it necessary that the diet prescribed should leave no residue which would mechanically irritate these ulcers and increase the tendency to hemorrhage and perforation. 3rd. The presence of tympanites, the tendency to which in typhoid is so great, largely increases the danger of hemorrhage and perforation, and therefore the diet must be such as to avoid this complication if possible. 4th. Another element, which enters in some cases, but not in all, namely, the presence of diarrhoea or constipation, which the dietician must take into account. 5th. The presence of fever means a greater waste of tissues, particularly of the protein elements. Vaughan, of Ann Harbor, in a recent paper before the American Association of Physicians has shown that fever results from the parenteral digestion of proteins. The diet must provide for this excessive waste. The first two elements in this four-fold problem demand of necessity a liquid diet. It is easy to take, easy to digest, and easy to assimilate, and leaves no residue; thus meeting the above mentioned requirements.

The solid food diet recommended a few years ago by a few enthusiasts has not met with general favor. It is wholly unscientific, as will be seen by considering the elements of the problem already mentioned, and has only one thing in its favor, the furnishing of a sufficient quantity of food to repair the waste. But the disordered digestion and diminished absorption preclude the probability of the patients getting the benefit of the additional amount ingested. To meet the second indication, we must not only have a diet which is liquid, but which does not become solid after entrance into the body, or else one which is easily liquided. To meet the third, we must have a diet which will not be liable to undergo fermentative changes in the intestine; and to meet the fourth we must have a diet rich in nitrogenous matter, one containing abundant proteid.

With one exception, milk would be the ideal diet to meet these conditions, for it is liquid, it contains a sufficient quantity of proteid in an easily digestible form, and it also contains carbohydrates, fats and salts. By many authorities milk is most highly recommended, but every one of them makes this statement about it. "If it disagrees with the patient it must be omitted." Personally I am opposed to milk as an article of diet in typhoid fever, for the following reasons: It becomes solid after it reaches the stomach; the impaired digestion, as outlined above, being unable to care for it; it undergoes putrefactive and fermentative changes, allows curds to pass into the intestines and to increase the tendency to hemorrhage and perforation, and to mechanically irritate. Clinically I have tested these so many times that I am convinced that the presence of milk in the diet of a typhoid fever patient will increase temperature and tympanites, and that the withdrawal of milk will diminish tympany and lower the temperature. Recently I consented almost against my judgment for a patient to have some ice cream, and found that within the next twenty-four hours his temperature was higher and tympanites greater. Undigested curds were found in the stool next day.

Buttermilk, when it can be taken by the patient, is the next best article of food, the small flocculent curds found in this not having the same disadvantage as the larger curds of sweetmilk. There are many patients who cannot take this without increasing tympany in which case it must be withdrawn. It takes also a larger quantity to supply the body's demands: 5½ oz. of buttermilk yielding 100 calories, while only 4½ of sweetmilk are required to yield the same amount.

Albumin administered with fruit juices, soups made of beef stock and flavored with vegetables, and thickened with rice or barley flour, beef blood squeezed from rare beefsteak, and the predigested foods sold in the market, meet the indications from a dietetic standpoint, being liquid, easily digested, and rich in proteids, and leave little residue. Various gruels, such as oatmeal gruel, rice gruel, barley gruel and certain of the malted preparations, may be given to furnish the carbohydrate elements, and also give variety to the patient, if he is not too sick to notice it. Gelatine, in the form of either chicken, wine, or fruit, jelly, or made with boiled custard in the form of Bavarian cream, is highly nutritious (one gram yielding the same number of calories as one of proteid) and has the advantage of not causing fermentative and putrefactive changes in the intestinal tract.

The great trouble with a merely liquid diet, other than milk, is the difficulty of administering a sufficient quantity to repair the excessive waste. Hence the great emaciation of most typhoid patients. A study of the caloric value of foods will help to overcome this difficulty. One ounce of peptonoids will yield 140 calories given every four hours will furnish 840 calories. in 24 hours. One albumin every 4 hours will yield 300 calories. These two will yield a total of 1140 calories. While this is greatly below the required quantity, it is very much greater than the daily requirement of proteid (450 calories.) Thus it furnishes the element needed, and the waste is largely in the heat-producing elements, carbohydrates and fats.

The broths are appetizing but not much more nutritious than albumin. It will take nearly 50 gallons a day to funrish the nutritive needs of the body.

This diet should be continued during the febrile period and for a few days thereafter. From then until ten days after the temperature has been normal the following articles may be added; milk, plain, or in the form of cream soups or shaken with eggs, egg soft boiled, soft scrambled, soft poached, scraped beef-ball, and thickened gruels. On the 10th day may be given, and from then on gradually may be added, broiled meats, soft rice. cereals, baked potatoes, and stale bread, in the order named.

The most important article of diet, however, in the case of typhoid is water, which must be given in great abundance, not less than two ounces every half hour. Of course the patient must not be waked from a quiet sleep for it. Water serves the threefold purpose of filling up the blood vessels and thus improving the circulation, of diluting the toxines, and increasing elimination. It may be given plain or carbonated, in the form of lemonade, or with some other fruit juice, or as weak tea, either cold or hot, which quenches thirst of the patient and is palatable.

So much for the internal use of water as an article of diet, but it has other important functions as a remedial measure in typhoid, for it is the remedy *par excellence* in this disease. It should be given internally, externally, eternally (and infernally.) The external use of water in typhoid fever depends upon its temperature more than upon the substance itself, for it is for the effect of cold usually, which is secured by the administration of water externally, that we employ it as a means, Perhaps, therefore, it would be best for us to recall the physiological action of cold when applied to the body. First, cold constricts through the stimulation of the vaso-motor nerves; later it paralyzes them with consequent dilation; it increases heat radiation, thus lowering the temperature of the body and in febrile conditions causes sedation. As applied in typhoid in the form of cold baths, its effect, therefore, is as above indicated. It stimulates the vaso-motor nerves, it improves the circulation, lowering the temperature, and causes a sedative effect on the nervous system.

The ordinary method of applying water externally in a case of typhoid fever is by means of cold bath either in the form of the tub bath or sponge bath. In private practice there are some difficulties in the use of the tub bath. As in the ordinary case equally good results can be obtained from the sponge bath, and as it is more easily given, it is the more practical of the two. There has been some effort recently to discredit the use of the bath, but the good effects obtained by it are too well known to shake the confidence in it, either on the part of the profession or the laity. Some have felt that the greatest benefit from it was the improvement in circulation, since it often occurs that the temperature is not materially lowered by it, and yet the patient shows improvement. But I must say that I believe that the increased heat radiation caused by it plays a large part in the benefits secured from it. Some of you may recall an experiment made by Wood some years ago, in which he demonstrated that the cerebral excitement as evidenced by delirium, tremor, etc., so often seen in typhoid, may be repeated by exposing an animal to severe heat, and that the mere withdrawal of heat causes the disappearance of these symptoms. I believe, therefore, that the reduction of temperature has much to do with the sedative effect following the cold bath.

The fact that the blood pressure is raised as one of the effects of the cold bath, makes it contraindicated in the case of hemorrhage.

The ordinary bath should be given at a temperature of from 40 to 50 degrees. Some patients do better when the temperature is from 70 to 80, and others when the water is tepid. In either case the effect of cold is obtained by the withdrawal of heat through evaporation. When the bath is given, gentle friction should also be used to assist the capillary circulation. Water is also used in typhoid in the form of colon irrigations. In addition to assisting elimination, it may be also used as a means of lowering the temperature in lieu of the bath.

Cold is also sometimes used on the head as an ice helmet, in cases of meningeal involvement, as the abdominal ice bag in cases of tympanites, and in hemorrhages.

The question of the specific sera in the treatment of typhoid is one which is engaging the attention of a great many to-day. Some very interesting results have been, and are being, obtained. Until very recently, the results have been so unsatisfactory that there was a tendency to look askance upon this method of treatment. There is no question in my mind but that the failures have been due to ignorance, that we have not learned the essential things for us to know in each case, and that as our knowledge on those points increases we will be able to get increasingly good results by this method. When a serum is needed a vaccine will not answer the purpose, and *vice versa.* A case which clinically appears to be typhoid, and yet which is not due to the bacillus typhosus, but to one of the allied groups, will not yield to a serum or vaccine prepared from Eberth's bacillus. It is necessary for us to know the infecting organism that the proper serum or vaccine may be used. In general, the following principle may be laid down, that anti-typhoid serum in a case of true typhoid should be administered when there is marked toxaemia. The best results are obtained, however, by using a vaccine prepared from the infecting organism. The resistance of the body begins to be raised six days after the injection. The initial dose of from 50 to 100 million should be repeated every two or three days, or at each marked rise in temperature, until immunity has been secured. After the administration of the vaccine there may be a slight rise of temperature, which is soon followed by a fall. But where the temperature is not changed materially the clinical symptoms show direct improvement, and the disease runs a mild course. Usually patients declare that they feel better shortly after the initial dose.

Walters and Easton, about two years ago, reported their results. In 32 cases of typhoid treated with vaccine there was a mortality of 3.2% present. Compare this with a mortality of 11% in 46 cases treated in the ordinary manner. Other observers have reported equally as good results. Personally I have had no experience as yet with this remedial measure, but a careful study of the reports leads me to believe that it is advisable to use a specific vaccine with the routine treatment of typhoid fever, combined in selected cases with the administration of an antitoxic serum. The object of this, of course, is to increase phagocytosis and bacteriolysis; since the pathogenic effects of the bacillus typhosus are probably due almost entirely to an endotoxin, and phagocytosis plays the most important part in the production of immunity. The administration of vaccine increase the opsonins, the agglutins, and the bacteriolysins.

Leaving out the special vaccine or serumtherapy, the treatment of typhoid fever resolves itself into an effort to maintain the body resistance, and to combat symptoms as they arise. Sometimes it is a nice point to decide what should be helpful and what meddlesome interference. Experience alone trains the judgment to determine what is right for each individual case; for we must never forget that we are treating the patient and not the disease.

